# Vitamin D Status of Residents in Taiyuan, China and Influencing Factors

**DOI:** 10.3390/nu9080898

**Published:** 2017-08-18

**Authors:** Xiaoning Yan, Jasmine S. Thomson, Ruibao Zhao, Ruifang Zhu, Zhaolin Wang, Na Zhang, Jane Coad

**Affiliations:** 1 Shanxi University of Traditional Chinese Medicine, 121, Daxue Street, Higher Education Park, Taiyuan 030619, Shanxi, China; 15034054587@163.com (X.Y.); zhaoruibao0604@126.com (R.Z.); 2 Massey Institute of Food Science and Technology, School of Food and Nutrition, Massey University, Private Bag 11222, Palmerston North 4442, New Zealand; j.coad@massey.ac.nz; 3 Shanxi Medical University, No. 56, Xinjian South Road, Taiyuan 030001, Shanxi, China; ruiruinuli@163.com; 4 Department of Traditional Chinese Medicine, The Second Hospital, Shanxi Medical University, 382, Wuyi Road, Taiyuan 030001, Shanxi, China; 13834627300@139.com; 5 Department of Hematology, Shanxi Hospital of Traditional Chinese Medicine, 16, Bingzhou West Street, Taiyuan 030012, Shanxi, China; zhangna166@163.com

**Keywords:** Vitamin D deficiency, metabolic syndrome, North China

## Abstract

High prevalence of vitamin D deficiency has been reported worldwide. Residents of Taiyuan, China, were predicted to be at high risk of vitamin D deficiency due to its high latitude, heavy air pollution, and cultural sun avoidance. This study investigated the vitamin D status of office workers, and explored the potential determinants of capillary 25-hydroxyvitamin D (25(OH)D) concentration as well as the relationship between 25(OH)D and metabolic syndrome. Two hundred participants, aged 20 to 80 years, were recruited. Capillary dried blood spot (DBS) 25(OH)D was measured; together with anthropometric (height, weight, and waist circumference), biochemical (serum lipid profile and fasting glucose) measures and a lifestyle questionnaire. Thirty-four percent of participants had 25(OH)D concentrations below 30 nmol/L, indicating deficient vitamin D status. Women’s 25(OH)D (median; 32.7 nmol/L (upper and lower quartile; 25.8, 43.8)) was significantly lower than men (44.0 nmol/L (32.3, 55.4)) (*p* < 0.01). Female gender, higher fasting glucose, and increased smoking (*p* < 0.05) were negatively associated with 25(OH)D concentration. However, there was no association found between metabolic syndrome (MetS) and 25(OH)D concentration and no significant difference in vitamin D status between men or women with MetS compared to healthy individuals. Vitamin D deficiency was common in urban residents of Taiyuan in winter and more so in women than men.

## 1. Introduction

Over the past few decades, China has experienced rapid economic development and urbanization, accompanied by social transition including nutrition and physical activity change. Lifestyles have shifted to increasingly unhealthy Western style diet behaviors [1], more sedentary jobs, and limited leisure time activity [2]. Not surprisingly, these trends have been accompanied by a rising prevalence of chronic diseases [3,4,5].

Vitamin D deficiency is associated with urbanization in terms of air pollution, increased sedentary occupations that result in most of the day being spent indoors, as well as, in China, a preference for light skin color and resultant sun avoidance [6]. The primary source of vitamin D is synthesis in the skin upon exposure to sunlight. However, the amount of solar ultraviolet-B (UVB) reaching the Earth’s surface is influenced by latitude, season, air pollution, and time of day [7]. For example, high latitude (>35° N) regions have considerably less UVB available for synthesis of pre-vitamin D in the winter [8]. Taiyuan, the capital city of Shanxi province in North China, is one of the main industrial bases of China. At latitude 38° N, there is low daily UVB radiation in winter, compounded by heavy air pollution from industry; this results in a significant number of residents being likely to have low exposure to UVB. 

The second source of vitamin D is diet, but there are few foods containing noteworthy amounts of vitamin D, except for some fatty fish, fish liver oils, and egg yolks. Intake of fatty fish and seafood is low in Shanxi province [9], which is situated inland. Additionally, there are limited vitamin D fortified foods available in China at present [6], and public awareness of vitamin D supplements is low [10]. The prevalence of vitamin D deficiency is relatively high in the general population; a recent multicenter study representative of five large cities in China found that the prevalence of vitamin D deficiency was 55.9% (serum 25-hydroxyvitamin D (25(OH)D) <50 nmol/L) in the urban population of Mainland China [11] according to the Endocrine Society Task Force recommendations [12].

Several research studies have demonstrated associations between vitamin D status and the prevalence of metabolic syndrome (MetS) [13]. MetS is a cluster of risk factors for cardiovascular events, including abdominal obesity, insulin resistance, hyperglycaemia, hypertension, and dyslipidaemia [3]. Metabolic syndrome is also associated with urbanization, for example urban dwellers have increasingly sedentary occupations and excess energy intakes [14]. Observational studies report that vitamin D deficiency is associated with risk factors for MetS including obesity [15] and diabetes [16] in Western populations. A limited number of studies have explored the relationship between vitamin D deficiency and MetS in Chinese populations [17,18,19]. Moreover, few other studies of vitamin D status in Chinese subjects have used the reference method for measuring 25(OH)D levels, liquid chromatography tandem mass spectrometry (LC-MS/MS), and these authors did not investigate MetS [11,20]. 

Therefore, the aim of the present study was to determine the vitamin D status of adults in Taiyuan during the winter season, using the reference method for analysis, and to explore determinants of vitamin D status. In particular, previously identified risk factors for vitamin D deficiency such as the presence of MetS criteria, Body Mass Index (BMI), age, gender, and lifestyle factors such as outdoor physical activity, alcohol consumption, and smoking were evaluated in this urban Chinese population.

## 2. Materials and Methods

The participants were recruited from individuals who attended the Health 100 Check-up Center for a regular health check-up during December 2013 and January 2014, which is the winter season in Shanxi, China. The study inclusion criteria were non-manual workers aged 20 to 80 years. Participants were excluded if they were diagnosed as having chronic liver disease, kidney disease, severe cardio-cerebral vascular disease, or cancer, or if they were pregnant. Since corporations often sponsor routine physical check-ups, many Health 100 Check-up Center patients may come from the same corporation. To avoid selection bias, only 10 to 15 volunteers were recruited per day to minimize concentrated sources of participants from the same corporation. The study received ethical approval from the Massey University Human Ethics Committee, Southern A (MUHEC) (Application number: 12/46). All participants gave written consent.

Qualitative data about health and lifestyle including smoking status, alcohol intake, daily physical exercise, and past medical history were collected during a face-to-face interview. Subjects were asked about their average daily and historical smoking habits and alcohol consumption. Answers on smoking habits were classified into “none” (never smoking), “light” (smoking less than 20 cigarettes per week), “medium” (more than 20 cigarettes per week), and “heavy” (20 cigarettes or more per day). Brot et al. [21] found a decrease, although non-significant, in serum 25(OH)D in smokers equivalent to our moderate and heavy categories. Answers on alcohol intake were classified into “none” (never drinking), “light” (drinking sporadically), “medium” (often drinking), and “heavy” (drinking every day); heavy drinking has long been associated with low serum 25(OH)D. Open-ended questions were asked about subjects’ time spent doing physical activity outdoors on an average day. Data were classified into “none”, “low”, “medium”, and “high”, where “none” was defined as no dedicated time for outdoor physical activity, “low” was defined as little dedicated time for outdoor physical activity, “medium” was doing exercise outdoors for less than one hour per day, and “high” was doing outdoor physical activity for more than an hour on an average day. An hour per day of outdoor activity was chosen to reflect the need for increased exposure to the sun to synthesize vitamin D in winter. However, we expected that little to no vitamin D would be synthesized during the study period, which experienced an average UVI of 2 and 3 and average temperature of −1 and 0 °C in December 2013 and January 2014, as reported on World Weather Online [22].

Participants fasted for 12 h before having venous blood samples taken from the median cubital vein. All blood sampling was handled by two nurses who had professional training. Biochemical markers included serum total cholesterol (TC), triglycerides (TG), high-density lipoprotein cholesterol (HDL), low-density lipoprotein cholesterol (LDL), and fasting glucose. The biochemical markers were analyzed by automatic biochemistry analyzer (Hitachi, 7600-020ISE) at the Health 100 Check-up Center in Taiyuan. 

The dried blood spot (DBS) method was used to collect whole blood for 25(OH)D analysis [23]. Briefly, finger prick capillary blood samples were collected on four pre-stamped circles on blood spot cards. Blood spots were air dried for at least 30 min before closing the cards, then sent in batches for analysis. 25-hydroxy vitamin D was analyzed by LC-MS/MS by the Department of Clinical Biochemistry, City Hospital, Birmingham, UK. This assay is accredited by the Vitamin D External Quality Assessment Scheme (DEQAS) and the laboratory has Clinical Pathology Accreditation. DBS has previously been validated against serum and whole blood samples for vitamin D measurement by tandem mass spectrometry (LC-MS/MS) [23]. However, because 25(OH)D is almost entirely excluded from the haematocrit fraction of the blood, DBS analytes need to be corrected for haematocrit concentrations. In the current study, the serum equivalent 25(OH)D concentrations were accounted for by using a standard haematocrit value of 0.4 [24]. The definition of vitamin D status in the current study was based on the Institute of Medicine (IOM) cut-offs where deficiency is serum 25(OH)D < 30 nmol/L; inadequacy is 30–50 nmol/L; and sufficiency is >50 nmol/L [25]. 

In addition, anthropometric data (height, weight, waist circumference) and blood pressure were collected by trained technicians from the Health 100 Check-up Center as part of the participants’ routine health check-up. Weight was measured to the nearest 0.1 kg and height and waist circumference (WC) were measured to the nearest 0.1 cm. Cut-off values indicating excess mass were body mass index (BMI) exceeding 24 kg/m^2^ defined as overweight, and BMI exceeding 28 kg/m^2^ as obese. The blood pressure of each participant was measured by a trained operator using an automatic sphygmomanometer (Omron HEM-7052, Omron Healthcare, Kyoto, Japan). 

The definition of MetS in the current study was based on the International Diabetes Federation (IDF) criteria and Working Group on Obesity in China (WGOC) recommendations. The criteria were: central obesity specific for Chinese people (waist circumference greater than or equal to 80 cm in females and 85 cm in males) [26], plus any two of the following: raised triglycerides (>1.7 mmol/L) or specific treatment for this lipid abnormality; reduced HDL-cholesterol (<1.03 mmol/L in men and <1.29 mmol/L in women) or specific treatment for this lipid abnormality; raised blood pressure (systolic blood pressure ≥ 130 mmHg or diastolic blood pressure ≥85 mmHg) or treatment for previously diagnosed hypertension; raised fasting plasma glucose (≥5.6 mmol/L) or previous diagnosis of type 2 diabetes [19].

All data were analyzed using SPSS version 21 (IBM Corporation, Armonk, NY, USA). Data distribution was tested for normality by the Kolmogorov-Smirnov Test. The data were expressed as means ± SD or median (inter-quartile range). The *t*-test was used to compare two groups of the data with normal distribution. The Manny-Whitney U test was performed to compare two groups of the data with non-normal distribution. A large multivariate model was used to show the factors with a significant association with 25(OH)D levels. Analyzed variables included age, BMI, MetS, WC, blood pressure, fasting glucose, TC, HDL, LDL cholesterol, triglycerides, smoking, drinking, and outdoor physical activity behaviors. MetS was entered into a multivariate model separate from its risk factors due to multicollinearity. Variable selection was conducted by in-and-out stepwise method. Multiple linear regression analysis was then conducted for the selected factors and controlled for gender. A *p*-value of less than 0.05 was considered to be statistically significant. 

## 3. Results

Characteristics of the study population are presented in Table 1. A total of 200 participants were recruited into the study. All 200 people provided finger prick capillary blood samples for 25(OH)D measurement. However, 11 participants did not have anthropometric measurements (height, weight, waist circumference, or blood pressure measurements) or venous blood samples taken. Another 25 participants ordered a different service package from the Health 100 Check-up Center, which did not measure HDL or LDL blood lipids. So, there were a total of 164 participants who had sufficient criteria measured to diagnose MetS. 

Almost two thirds of participants were women (63.5%) and the median age was 48 years. The median (interquartile range) concentration of 25(OH)D was 35.4 (27.9, 47.1) nmol/L and was significantly lower in women than men. Almost one third (29.9%) met the criteria for MetS and 13.8% were calculated to have obesity (BMI exceeding 28 kg/m^2^); both MetS and obesity were more common in men (29.3%, 24.6%) than women (7.5%, 20.6%). 

All 200 participants had DBS 25(OH)D measured. The median values of 25(OH)D_2_, 25(OH)D_3_, and total 25(OH)D in males and females are presented in Table 2. There were significantly higher total 25(OH)D and 25(OH)D_3_ concentrations in men than women. However, the difference in 25(OH)D_2_ concentrations by gender was not statistically significant (*p* = 0.89). 

Using IOM vitamin D deficiency criteria and corrected 25(OH)D concentrations, 33.5% of participants were classified as deficient (<30 nmol/L); 44.5% were inadequate (30–50 nmol/L); and 22.0% were sufficient (≥50 nmol/L) [25]. When stratified by gender, 40.9% of women were deficient and 20.5% of men were deficient.

There was not considered to be a statistically significant difference in 25(OH)D concentration in participants with MetS compared to healthy individuals; *p*-value was 0.05, but gender was a confounding factor. When stratified by gender, there was no significant difference in 25(OH)D between women or men with MetS compared to healthy individuals (*p* = 0.41, *p* = 0.40), respectively. In the multiple linear regression analysis, when gender was controlled for, there was no significant association between MetS and 25(OH)D concentration. 

Multiple linear regression analysis was performed with 25(OH)D as the dependent variable and controlled for gender. In Table 3, the results of the multiple linear regression analysis are presented; gender, fasting glucose, and smoking were the independent factors predicting 25(OH)D. Being female, increased fasting glucose, and increased cigarette consumption had negative association with 25(OH)D concentration. 

## 4. Discussion

The findings from this study suggest that vitamin D deficiency is common in office workers in Taiyuan, China during the winter season according to IOM criteria [25]. The current study found that 40.9% of women and 20.5% of men were vitamin D deficient in Taiyuan during winter. Prevalence was higher than reported recently in China in a nationally representative sample of adults over 60 years, in which 12.0% of women and 7.8% of men were classified as deficient over all seasons [27]. However, prevalence in the current study was lower than peak vitamin D deficiency which occurred in spring, where 50% of women and 40% of men were deficient [27]. 

Other consensus bodies such as the Endocrine Society Task Force [12] and panelists at a Central European vitamin D conference in Poland [28] recommend higher serum 25(OH)D concentrations to define vitamin D status. These bodies define deficiency as serum 25(OH)D <50 nmol/L, and sufficiency as >75 nmol/L. These recommendations are more commonly reported in studies on the Chinese population [11,18,20,29]. If the Endocrine Society Task Force definitions were used for the current study, vitamin D deficiency prevalence would be 78%, which is much greater than the incidence of vitamin D deficiency reported in healthy Chinese adults (29–60% prevalence) [11,18,20,29]. Nonetheless, the strength of the evidence relating the Endocrine Society Task Force cut-offs with increased risks of related morbidities is unclear [30].

The median 25(OH)D concentrations (men: 44.0 nmol/L and women: 32.7 nmol/L) found in the current study were similar to other studies conducted at high latitudes in China during winter [31,32]. Woo et al. [31] found women of childbearing age in Beijing (39° N) had 29 nmol/L 25(OH)D, and Zhou and colleagues [32] found a healthy elderly population in Shenyang (41°–43° N) had a mean plasma 25(OH)D concentration of 31 nmol/L. 

Vitamin D deficiency in Taiyuan might be affected by environmental factors. For example, high latitude (>35° N) regions have considerably less UVB available for the synthesis of pre-vitamin D in the winter [8]. The zenith angle of the sun is increased and more UVB is absorbed by the earth’s ozone layer, which leads to less UVB reaching the earth’s surface, thereby affecting the synthesis of 25(OH)D_3_ [7]. However, other studies in Northern China found similarly low 25(OH)D concentrations compared to the current study during the summer months [20,33]. 

Taiyuan is traditionally an industrial city and air pollution has been high for many years. Dust haze occurs more often during the winter; it was reported that in the winter months, dust haze occurred on average up to 14 days per month [34]. The dust haze mostly occurred from 8 a.m. to 1 p.m., which significantly decreased the surface solar radiation intensity compared with no-haze days [34]. Whilst some studies indicate an association between living in an urban environment compared to rural areas [27], our results are similar to a mainly rural population, Linxian, also within Shanxi province [29]. Lin et al. [29] found an average 25(OH)D concentration of 31.7 nmol/L in the mostly subsistence farmer population expected to spend a large amount of time outdoors.

On the other hand, lifestyle factors may have a greater impact on 25(OH)D levels than latitude or residential environment. A number of studies, including two in China, indicate that individuals living in coastal cities, even those at high latitudes such as Dalian (39° N), have better vitamin D status than those in inland cities such as Beijing (39.5° N) and Urumqi (43.5° N) [11,20]. The postulated reason for this was fish consumption [11]. Taiyuan is located several hundred kilometers inland and fatty fish are not commonly consumed [9]. Although, interestingly, 13 participants of the current study had very high 25(OH)D_2_ concentrations, even though they reported that they did not take vitamin D_2_ supplements. Possibly, these individuals consumed foods containing high vitamin D_2_, for instance sun-dried mushrooms are a popular food source of D_2_ [35]; however, dietary intake assessment was not conducted in the present study. 

Median 25(OH)D was significantly higher in men than women and 25(OH)D concentration was positively associated with male gender. Similar findings have also been reported in some [27,29,33], but not all previous studies [36,37]. One of the reasons for this gender difference could be different lifestyles of men and women. Women are more likely to try to reduce sun exposure by covering up with clothing, sunscreen, and umbrellas, as they have a greater preference for light skin [38]. However, Yan et al. [36] found the opposite, that 25(OH)D levels were higher in women than men in Shenyang, China. The authors also proposed that this could be due to different lifestyles between genders, and that women in Shenyang spent greater time in outdoor activities, such as shopping in the open markets and in their yards talking to neighbors, compared to men [36]. 

Comparison of the current study with others is difficult, since in different studies different cut-off values for vitamin D deficiency have been reported, different samples have been collected, and different analytical methods have been used. For instance IOM [27], Endocrine Society Task Force [11,18,20,29], and other guidelines [31,32] have been used to classify the vitamin D status of various populations. In previous studies, plasma [32,36,37] or serum samples [11,17,18,20,27,29,31,39,40] were collected; the use of DBS collection in the current study was addressed by correcting for haematocrit concentrations to adjust concentrations to a serum equivalent value [24]. Finally, researchers have used different methods to analyze 25(OH)D samples, particularly in earlier studies. In the current study, the reference method for 25(OH)D determination, LC-MS/MS, was used, which has the added advantage of determining both D_2_ and D_3_ forms. In contrast, most others have used radio-immunoassay [31] or enzyme immunoassay to assess 25(OH)D [29]. In a few studies LC-MS/MS [11,20] was used, however, no studies reported DBS collection on the Chinese population for direct comparison. 

The prevalence of MetS in the current study was 29.9%. This is higher than that found by a recent meta-analysis which reported that prevalence of MetS in urban individuals in Mainland China was around 24.9% [4], although this not as high as the urban population of Qingdao (Northern China) which had a prevalence of 36.7% [5] based on IDF criteria. Previously, research has suggested that MetS is more prevalent in urban compared to rural residents and in Northern China compared to Southern China [41]. 

In the current study, MetS was not found to be associated with 25(OH)D levels, although fasting glucose was found to have a small negative association with 25(OH)D levels. Others found significant inverse associations between 25(OH)D and MetS [17], fasting glucose [17,18,40], fasting insulin [18,40,42], Homeostasis Model Assessment (HOMA)-IR [40,42], HOMA-B [40], triglycerides [17,18], waist circumference [17], blood pressure [17], and positive association with HDL cholesterol in Chinese adults [17,18]. Some authors indicate there may be ethnic differences in the association of fasting glucose with serum 25(OH)D [43]. Possible mechanisms for an association between 25(OH)D and glucose homeostasis include effects on β-cell function and insulin sensitivity, although there is no evidence for causality [16]. 

Smoking was also found to have a weak negative association with 25(OH)D levels. Other studies have also found smoking to have a significant negative association with serum 25(OH)D [21,44]. The mechanisms whereby smoking is related to decreased vitamin D metabolites remain to be elucidated, but it has been postulated that smoking decreases hepatic 25 hydroxylase (CYP2R1) and therefore lowers serum 25(OH)D [21]. 

A strength of the current study is that 25(OH)D concentrations were measured with LC-MS/MS [11]. However, a limitation of the current study was not evaluating vitamin D dietary intake; a diet diary or food frequency questionnaire on vitamin D-rich food consumption would have been useful. A further limitation in the current study was the inadequate depth obtained by some questions in the lifestyle questionnaire. Specifically, the classifications of outdoor physical activity gave insufficient information on sunlight exposure independent of physical activity. Future research might include an investigation of 25(OH)D concentrations in Taiyuan adults during spring which is likely the period when the nadir in 25(OH)D concentrations are reached, or in summer, representing the likely peak 25(OH)D concentrations. Following the finding that vitamin D deficiency is common in the current study population of office workers, future research on more vulnerable population groups such as young children, women of child-bearing age and pregnant women, and the elderly residents of Taiyuan is required. If further studies confirm these findings, then public health measures such as food fortification may be needed to address the low vitamin D status in Taiyuan.

## 5. Conclusions

In conclusion, vitamin D deficiency was common in non-manual workers in Taiyuan, China during the winter season. The vitamin D status of women was lower than that of men. Female gender, increased fasting glucose, and increased smoking were significant determinants for 25(OH)D concentration. 

## Figures and Tables

**Table 1:** Characteristics of the participants.

Characteristics	*n*	Men	Women	*p*-Value
Age (y) (median, IQR)	200	49.0 (31.0, 60.5)	48.0 (35.0, 55.0)	0.51
BMI (kg/m^2^) (mean ± SD)	189	25.9 ± 2.8	23.6 ± 3.1	<0.01
WC (cm) (median, IQR)	189	89.8 (82.8, 96.8)	77.1 (73.6, 82.8)	-
SBP (mmHg) (median, IQR)	189	132 (118, 143)	119 (110, 131)	<0.01
DBP (mmHg) (median, IQR)	189	82 (75, 92)	73 (67, 80)	<0.01
TC (mmol/L) (mean ± SD)	189	5.08 ± 0.87	4.95 ± 0.10	0.36
TG (mmol/L) (median, IQR)	189	1.78 (1.15, 2.55)	1.15 (0.77, 1.76)	<0.01
HDL (mmol/L) (median, IQR)	164	1.23 (1.11, 1.33)	1.46 (1.29, 1.65)	<0.01
LDL (mmol/L) (mean ± SD)	164	3.29 ± 0.76	3.12 ± 0.81	0.17
FG (mmol/L) (median, IQR)	189	6.01 (5.78, 6.24)	5.67 (5.31, 6.07)	<0.01
25(OH)D (nmol/L) (median, IQR)	200	44.0 (32.3, 55.4)	32.7 (25.8, 43.8)	<0.01

Where; y: years of age; IQR: inter-quartile range; SD: standard deviation; BMI: body mass index; WC: waist circumference; SBP: systolic blood pressure; DBP: diastolic blood pressure; TC: total cholesterol; TG: triglyceride; HDL: high density lipoprotein cholesterol; LDL: low density lipoprotein cholesterol; and FG: fasting glucose.

**Table 2:** Mean values of 25(OH)D in men and women.

Vitamin D	Men (*n* = 73)	Women (*n* = 127)	*p*-Value
T25(OH)D(nmol/L) (median, IQR)	44.0 (32.3, 55.4)	32.7 (25.8, 43.8)	<0.01
25(OH)D_3_ (nmol/L) (median, IQR)	37.9 (28.2, 48.9)	27.2 (21.8, 37.7)	<0.01
25(OH)D_2_ (nmol/L) (median, IQR)	3.60 (2.80, 4.95)	3.30 (2.80, 5.70)	0.89

Where; IQR: inter-quartile; 25(OH)D: 25-hydroxyvitamin D; 25(OH)D_3_: 25-hydroxyvitamin D_3_; 25(OH)D_2_: 25-hydroxyvitamin D_2_.

**Table 3:** Multiple linear regression analysis for 25(OH)D concentration.

Variables	B	SE	*p*-Value	*F*-Value
Intercept	58.23	16.2	<0.01	12.9
Gender	−12.12	3.30	<0.01	13.5
WC	0.24	0.14	0.09	2.91
FG	−2.32	0.91	0.01	6.48
Smoking	−4.04	1.88	0.03	4.60

Where; B: beta coefficient; SE: standard error; WC: waist circumference; FG: fasting glucose; gender was entered into the model as categorical variables coded men = 1, and women = 2, and smoking was coded as none = 1, light = 2, medium = 3, and heavy = 4.
